# List of reviewers 2025

**DOI:** 10.1192/bji.2026.10122

**Published:** 2026-11

On behalf of the editorial board, the Editor-in-Chief would like to thank the following for their contribution as peer reviewers in the period from January 2025 – December 2025. Their anonymous work for the journal forms the foundation to its success and is highly appreciated.

Abdel Aziz, Karim

Abdelgawad, Ahmed

Ahmed, Hamdia Mirkhan

Alarcon, Renato

Al-Ayyat, Dania

Al-Dhabbhari, Fatma

Ali, Mohamed

Allam, Mohamed

Al-Salihy, Zerak

Al-Sinawi, Hamed

Aluh, Deborah

Al-Uzri, Mohammed

Assar, Ahmed

Balcioglu, Yasin Hasan

Barber, Sarah

Barrett, Elizabeth

Bass, Nicholas

Basu, Aniruddha

Belalcazar Vivas, Valentina

Berrisford, Giles

Bhad, Roshan

Budden, Emily

Butner, Jenna

Carrà, Giuseppe

Castellana, Matthew

Chadda, Rakesh

Chakraborty, Nandini

Chawla, Nishtha

Christodoulou, Nikos

Courtenay, Ken

Dada, Mobolaji Dada

Daradkeh, Tewfik

Das, Anindya

Ebrahim, Walid

Egbe, Catherine

Eilinghoff, Luisa Marie

El-Gabry, Dina

Elhusein, Bushra

Elkholy, Hussien

El-Khoury, Joseph

El-Maraghy, Manal

ELNahas, Gihan

Elnemais, Michael

Elshahed, Mostafa

Farooq, Saeed

Fawzi, Tatjana

Fenton, Sarah-Jane

Foulkes, Lucy

Furst, Mary Anne

Gazdag, Gábor

Gehlot, Sanjay

Ghosh, Abhishek

Gibbons, Rachel

Giotakos, Orestis

Grover, Sandeep

Hall, Katie

Hannon, Kayla

Heinz, Andreas

Huby, Guro

Hudnall, Matthew

Hughes, Peter

Humphreys, Keith

Hyland, Declan

Inagawa, Takuma

Jalloh, Abdul

Jarukasemkit, Setthanan

Javaid, Syed

Javed, Afzal

Jenkins, Rachel

Kabir, Russell

Kallivayalil, Roy Abraham

Kar, Sujita

Khaled, Salma

Khamker, Nadira

Khan, Zarshed

Kisely, Steve

Kokonya, Donald

Kotsis, Konstantinos

Krishnan, Vijay

Kumar, Mahesh

Kumar, Nimisha

Lada, Georgia

Large, Matthew

Llorca-Bofí, Vicent

Loch, Alexandre

Luu, Trang

Lyndon, Stanley

Manohar, Harshini

Mansour, Omar

Martiadis, Vassilis

Matínez, Pablo

Mirza, Hassan

Morsy, Mahmoud

Mubarak, Ahmed

Ndetei, David

Okpaku, Sam

Onu, Justus U.

Onyechere, Udoka

Opondo, Philip

Pal, Arghya

Peritogiannis, Vaios

Qassem, Tarek

Qassem, Tarik

Rajan, Tess

Ramzy, Ramez

Raouf, Nabila

Rivera, Guillermo

Rooper, Cath

Salvador-Carulla, Luis

Samara, Myrto

Sarkar, Siddharth

Seleem, Mohammad A.

Sen, Mahadev Singh

Skuse, David

Smith, Andrew

Stavert, Jill

Swanepoel, Annie

Tadros, George

Tan, Marcus

Tangjittiporn, Thiraporn

Telesia, Laurence

Torales, Julio

Tran, Phuoc

Ularntinon, Sirirat

Wadoo, Ovais

Watkins, Lance

Woodruff, Peter

Xenaki, Lida-Alkisti

Zavrou, Marianna

Zixin Li, Lambert

